# Stool sampling and DNA isolation kits affect DNA quality and bacterial composition following 16S rRNA gene sequencing using MiSeq Illumina platform

**DOI:** 10.1038/s41598-019-49520-3

**Published:** 2019-09-25

**Authors:** Petra Videnska, Kristyna Smerkova, Barbora Zwinsova, Vlad Popovici, Lenka Micenkova, Karel Sedlar, Eva Budinska

**Affiliations:** 1 RECETOX, Faculty of Science, Masaryk University, Kamenice 5, 625 00 Brno, Czech Republic; 2 Department of Biomedical Engineering, Brno University of Technology, Technicka 12, Brno, Czech Republic

**Keywords:** Next-generation sequencing, Bacterial genes, Metagenomics

## Abstract

Many studies correlate changes in human gut microbiome with the onset of various diseases, mostly by 16S rRNA gene sequencing. Setting up the optimal sampling and DNA isolation procedures is crucial for robustness and reproducibility of the results. We performed a systematic comparison of several sampling and DNA isolation kits, quantified their effect on bacterial gDNA quality and the bacterial composition estimates at all taxonomic levels. Sixteen volunteers tested three sampling kits. All samples were consequently processed by two DNA isolation kits. We found that the choice of both stool sampling and DNA isolation kits have an effect on bacterial composition with respect to Gram-positivity, however the isolation kit had a stronger effect than the sampling kit. The proportion of bacteria affected by isolation and sampling kits was larger at higher taxa levels compared to lower taxa levels. The PowerLyzer PowerSoil DNA Isolation Kit outperformed the QIAamp DNA Stool Mini Kit mainly due to better lysis of Gram-positive bacteria while keeping the values of all the other assessed parameters within a reasonable range. The presented effects need to be taken into account when comparing results across multiple studies or computing ratios between Gram-positive and Gram-negative bacteria.

## Introduction

The gut microbiome plays a key role in shaping human health and has been the subject of an increasing number of studies in the context of disease development, diagnostics and treatment. Important progress has been made especially in investigating uncultured bacteria, which constitute the main part of the gut microbiome and were previously difficult to characterize with standard techniques such as cloning, Sanger sequencing or Denaturing Gradient Gel Electrophoresis (DGGE)^1,2^. Next generation sequencing (NGS) provides new and more detailed means to study the human microbiome and helps uncovering its impact on the human immune system development^3–5^, or on the development of chronic diseases^6,7^. However, human microbiome is very dynamic and can change rapidly in response to many factors such as diet, antibiotic use, lifestyle or environment^8–16^. Many diseases were associated with a phenomenon called dysbiosis – microbial imbalance. Unfortunately, due to the huge microbiome variability it is very difficult to define a normality baseline for an individual. To extract disease-relevant information and generate new or confirm existing biological hypotheses, large cohort microbiome studies are needed. These studies face multiple challenges with the microbiome sampling. First, successful compliance of participants with the established protocol demands both motivation and an easy sampling procedure. Especially, sampling of the stool at home can induce a “yuck effect” and positive education and uncomplicated sampling workflow can significantly decrease the number of study drop-outs^17,18^.

Another major problem is the large variability of methodological approaches employed by different microbiome studies. The final composition of bacteria as assessed by sequencing the 16S rRNA gene is influenced by many factors: sampling method^19–22^, sample storage conditions^20,22–29^, DNA extraction^8,21,22,26,30–39^, primers targeting different parts of the 16S rRNA gene^40,41^ and data analysis^42^. All of these factors may lead to the misinterpretation of changes in the microbiome and thus hamper direct comparisons of results between individual studies^43–45^. These technical problems, along with an as yet unknown gut microbiome diversity in the healthy population, lead to challenges in the implementation of metagenomics into cohort studies and, in consequence, delay the translation of the knowledge to clinical practice.

Most studies focused on the technical factors influencing the assessment of bacterial composition often provide only a description of the observed differences on a limited number of samples, while the comparison of the effect sizes of these factors, or combination thereof remains unexplored. The effect of sampling was previously described with respect to storage conditions (such as temperatures^20,23,26,28,29^, periods at room temperature^20,24^ or a presence and type of stabilizer^19,21,22,27,28^). None of these studies reported on the volunteers’ compliance or the differences in preprocessing steps specific to different sampling kits. Multiple studies describe the effect of stool homogenization prior DNA extraction^25,46^, but they only report its overall effect on the interindividual variation, without quantifying this effect at different bacterial taxon levels.

The DNA extraction method was highlighted as a critical factor influencing the observed bacterial composition^39,47^. Commercially available extraction kits use different lysis procedures such as enzymatic, chemical or mechanical bacterial cell disruption methods. Generally, the combination of enzymatic and mechanical disruption is recommended as more effective in the lysis of Gram-positive bacteria^8,22,26,34,35,37,39^. However, these DNA extraction comparison studies are limited to a rather small number of individuals (from 2 to 9) and none of them compared the kits in terms of DNA yield and quality, presence of PCR inhibitors, the human to bacterial DNA ratio, the efficiency of Gram-positive bacteria cell wall lysis and the observed bacterial composition at different taxa levels all at once.

The aim of our study was therefore to perform systematic assessment of effect of sampling and DNA isolation kits and their combinations on a full range of parameters of bacterial DNA quality, bacterial diversity and composition, with respect to user acceptance.

## Results

We analyzed stool samples from sixteen volunteers. Each volunteer collected the samples from the same stool sample using three different sampling kits (SK): a stool container (SK1); a flocked swab (SK2) and a cotton swab (SK3). The DNA was extracted using two isolation kits PowerLyzer PowerSoil DNA Isolation Kit (PS) and QIAamp DNA Stool Mini Kit (QS) (see Methods), totaling 96 samples for the analysis.

### Evaluation of user acceptance of the sampling kits

The participants were asked to select the best and the worst kit based on their ease of manipulation including the time spent using it. All 16 volunteers selected the stool container as the easiest to use and 13 out of 16 (81.25%) volunteers indicated the flocked swab as the worst sampling kit. We believe that the manipulation with cotton and flocked swabs is uncomfortable due to the small size and the necessity to insert the swab stick back into the tube without touching the tube wall. On the contrary, the stool container is easy to manipulate even for people with reduced motoric skills. In addition, the flocked swab is designed for sampling of liquid samples and the solid stool samples do not adhere on its synthetic fibers.

### The effect of sampling and DNA isolation kits on the bacterial gDNA quality

#### DNA yield, purity and integrity

**Figure 1:**
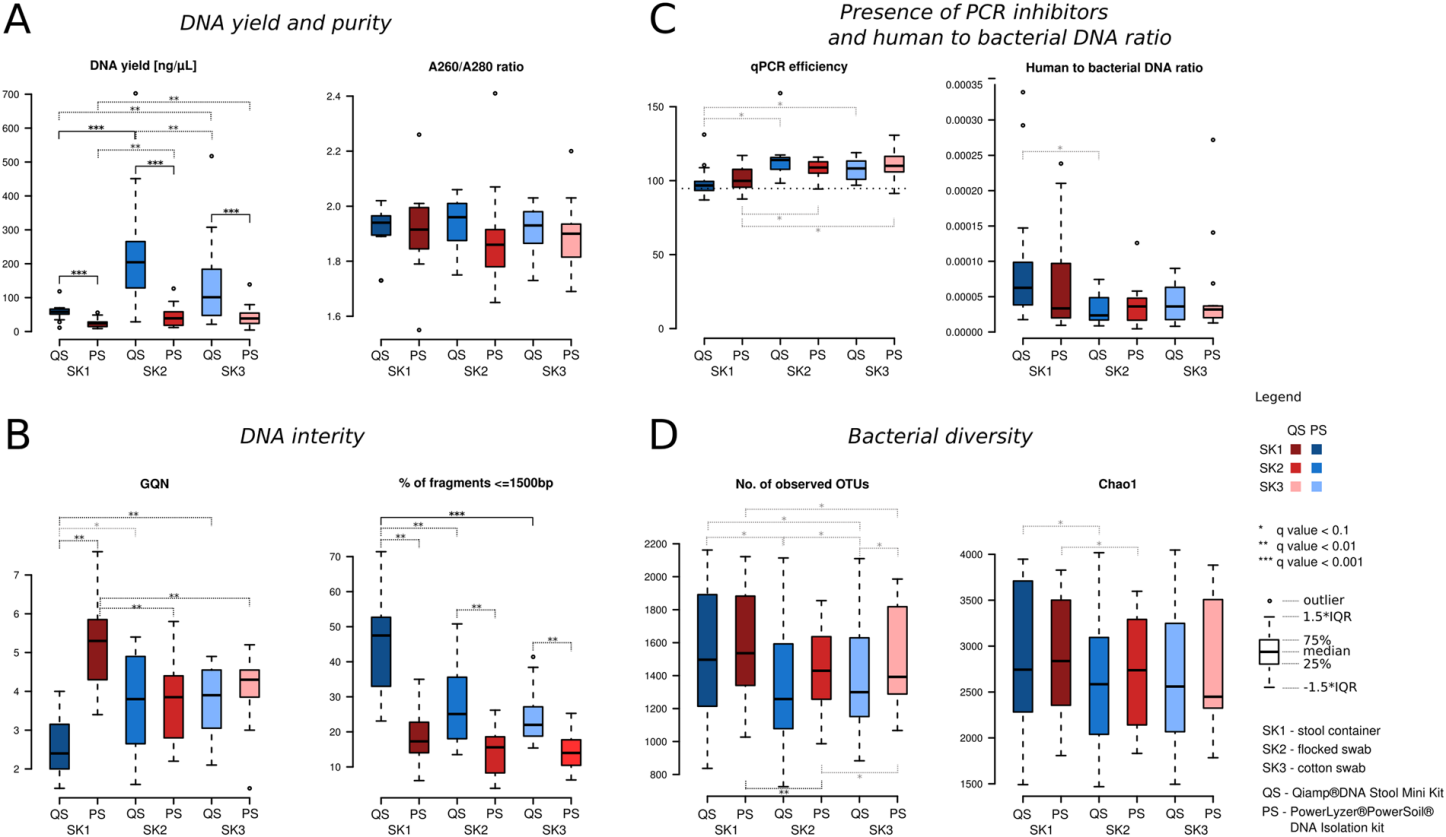
Comparison of sample DNA quality and diversity using different sampling and isolation kits. (**A**) DNA yield and purity comparison. ^d^the samples were five times diluted prior the DNA extraction (see Methods); (**B**) DNA integrity comparison; (**C**) Presence of PCR inhibitors and human to bacterial DNA ratio comparison. Horizontal dotted line represents median efficiency value of the positive control; (**D**) Bacterial diversity comparison.

Significantly higher DNA yields were obtained with the QS isolation kit, regardless of the sampling kit used (q < 0.01) (Fig. 1, Supplementary Table S1). The median values of the A260/A280 ratio (the measure of purity of DNA) were well within the expected range (1.8–2) and did not differ significantly between the DNA isolation kits or between the sampling kits (Fig. 1, Supplementary Table S1).

The DNA integrity was determined using the GQN measure (on a scale from 1 to 10; low GQN indicates strongly degraded gDNA sample) and the proportion of short fragments (≤1500 bp; the larger the proportion the more degraded gDNA). We observed interaction effects of isolation and sampling kit for both DNA integrity measures. We found significantly lower proportion of short fragments when using the PS isolation kit (Fig. 1, Supplementary Table S1) and this difference was much larger when the stool container was used for sampling. There was no difference in GQN measure between the isolation kits when cotton or flocked swabs were used. However, for stool container samples, the QS kit provided much lower GQN values compared to the PS kit. These results point to worse DNA integrity for the QS isolation kit compared to the PS isolation kit mostly when stool container is used for sampling.

#### Presence of PCR inhibitors

The presence of PCR inhibitors in the samples decreases the sensitivity of the PCR reaction and even can lead to the impossibility of amplification of the selected region of 16S rRNA. It is usually measured by median efficiency values estimated from inhibition plots. Ideally, the efficiency should be 100%, meaning the template doubles in each cycle. Usually, the efficiency within 90–110% range is considered acceptable, where lower efficiency is caused by non-optimal reagent concentration or lower enzyme quality, while higher efficiency values are caused by the presence of PCR inhibitors. In our data, the efficiency values ranged from 96.7% to 114.0% (Fig. 1, Supplementary Table S2). In each of the isolation/sampling kit combinations, there were minimum two samples which exceeded the efficiency of 110%. The efficiency values of all isolation/sampling kit combinations, except for stool container samples after DNA isolation with the QS kit, were significantly increased compared to control samples without PCR inhibitors (efficiency_med_ = 94.7%). No difference in efficiency values was observed between the isolation kits. The samples from stool containers (regardless of the isolation kit used) contained less PCR inhibitors in comparison to all other sampling/DNA isolation kit combinations (significantly lower efficiency, Supplementary Table S2). We hypothesize that this sampling kit effect is due to the sample dilution step prior to the DNA isolation step.

#### Human to bacterial DNA ratio

In all samples, the quantity of human DNA was lower than that of the bacterial DNA (ranging from 2947x to 221239x, median 29369x, see Fig. 1, Supplementary Table S2). No difference was found between sampling/isolation kit combinations in terms of human to bacterial DNA ratio, except for the increased ratio in the stool container compared to flocked swab samples after isolation with the QS kit (q = 0.03).

### The effect of sampling and DNA isolation kits on bacterial diversity and composition

#### Bacterial diversity

In total, 96 stool samples were sequenced. The number of reads after quality filtering and removal of chimeras ranged from 27680 to 67809, with median of 46192. We assessed the bacterial diversity using the number of observed OTUs and the Chao 1 diversity metric (Fig. 1, Supplementary Table S1). Overall, both diversity measures were independent of the DNA yield in all sampling/DNA isolation kit combinations.

While there was no difference in Chao 1 measure between the isolation kits, the number of observed OTUs was significantly increased after isolation with the PS kit, but only for cotton swab samples (q-value = 0.029). When comparing diversity measures between the sampling kits within each isolation kit separately, the stool container resulted in significantly higher number of observed OTUs in both DNA isolation kits (Fig. 1, Supplementary Table S1). In addition, we observed significantly higher number of OTUs in flocked swab samples compared to cotton swab samples after DNA isolation with the PS kit (q-value = 0.04) and significantly lower number of OTUs in flocked swab samples compared to cotton swab samples after DNA isolation with the QS kit (q-value = 0.09). For the Chao 1 diversity metric, significant differences were found in stool container samples compared to flocked swab samples in both PS and QS isolation kits (q = 0.04 and q = 0.09, respectively).

#### Bacterial composition

We identified 12,948 OTUs belonging to 13 bacterial phyla.

**Figure 2:**
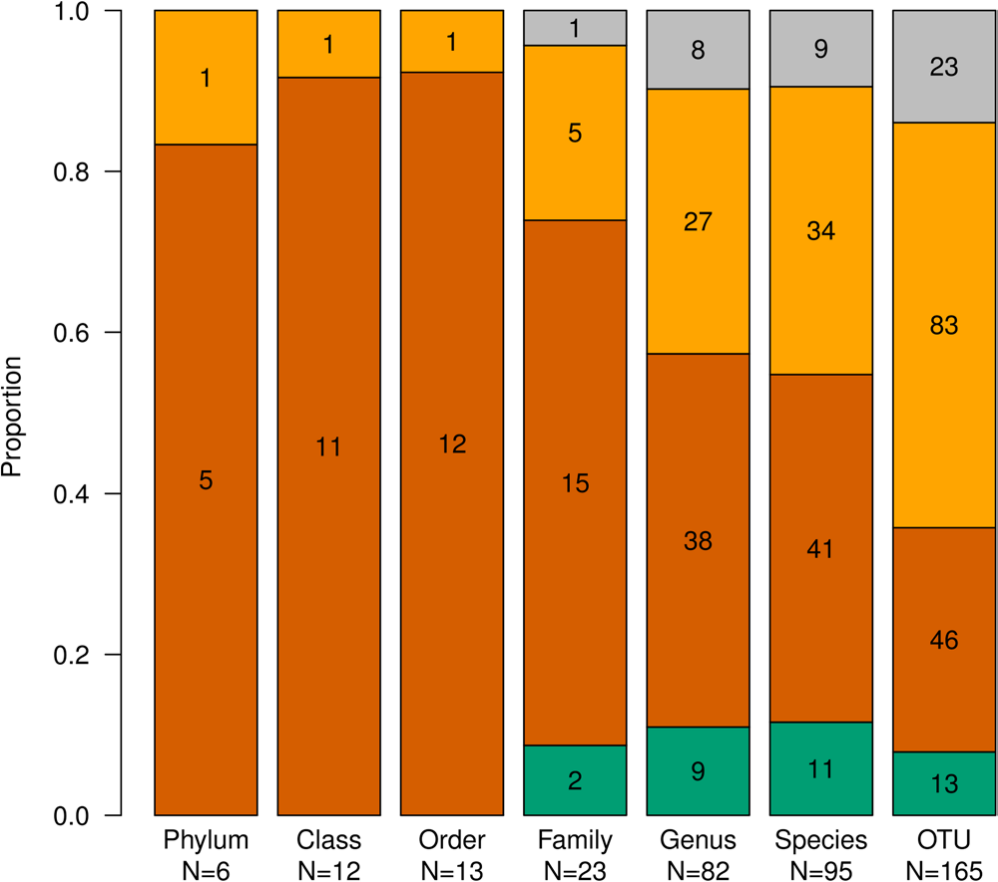
The proportion of taxa significantly affected by sampling or isolation kit at different taxonomical levels. Proportion of the tested taxa significantly affected by the sampling kit only (green), by the isolation kit only (dark yellow) and by both sampling and isolation kit (brick red). Grey indicates taxa not affected by sampling or isolation kit. The significance level was chosen at FDR < 10%, only taxa that met the selection criteria (maximum abundance >1%) were tested.

**Figure 3:**
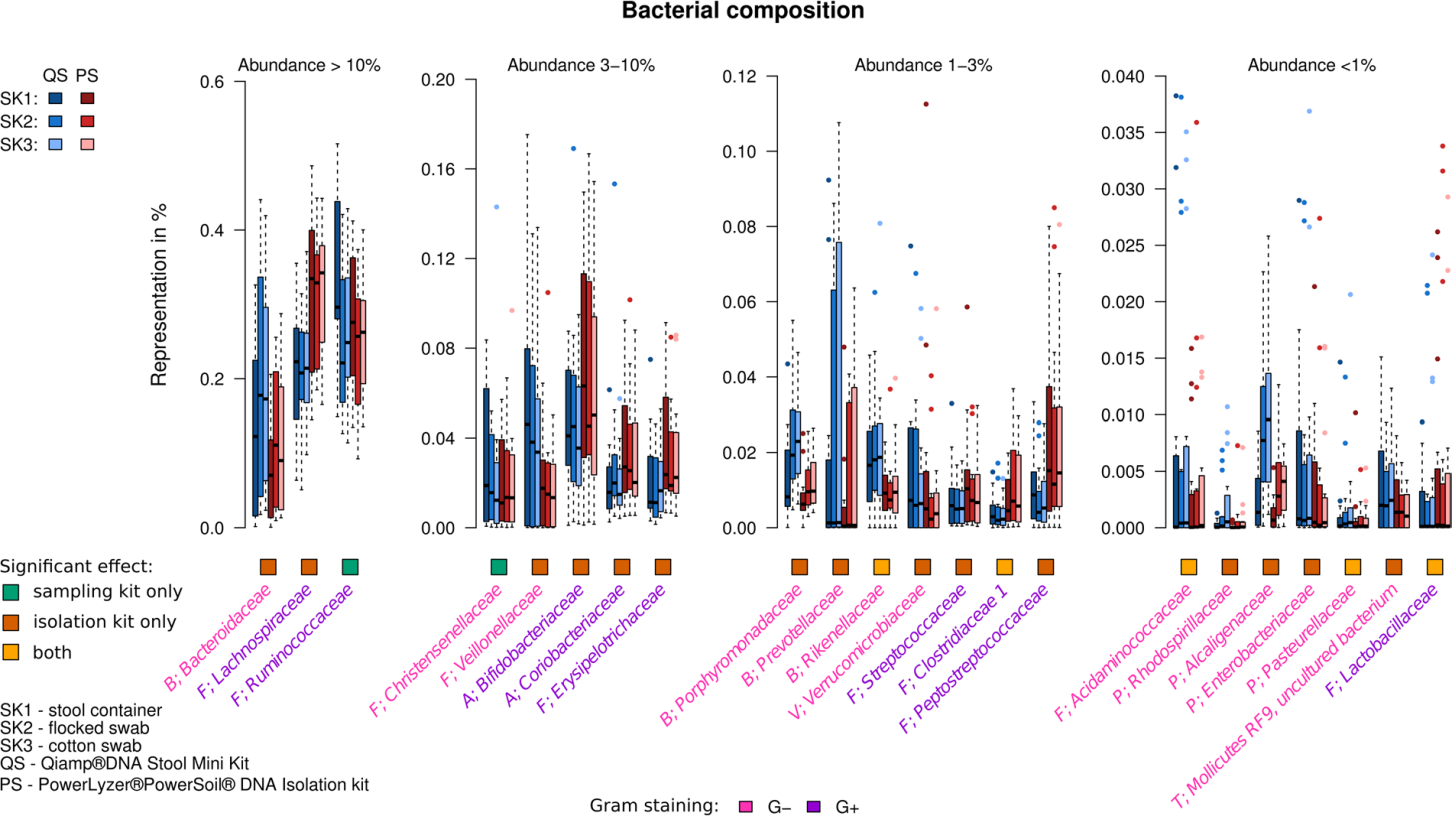
Distributions of relative abundances of significantly affected taxa at family level. Four graphs represent families divided according to third quartile of their abundance. Only taxa that passed the filtering criteria (maximum abundance >1%), significantly affected by isolation or sampling kit are shown. The colored squares below the graph indicate whether the family was affected significantly by the sampling kit only, the isolation kit only or both.

In order to quantify the effect of the sampling and isolation kits on bacterial composition, we performed mixed linear regression on each taxon that passed the filtering criteria (maximum abundance across all samples ≥1%) at all the seven taxonomical levels (phylum, class, order, family, genus, species, OTUs) separately. Interestingly, the proportion of taxa significantly affected by isolation or sampling kit differed between taxonomical levels (Fig. 2). The choice of sampling or DNA isolation kit affected 100% of taxa at phylum, class and order levels, and had decreasing trend from family to OTU level. The effects of sampling and isolation kits on the ten most abundant taxa at different taxa levels are summarized in Table 1 (see Supplementary Tables S3–S8 for complete results), the composition of significantly affected families is shown in Fig. 3. Overall, the choice of the isolation kit affected the abundance of more taxa than the choice of the sampling kit. In most of the cases where the taxa was affected by both factors, the p-values associated with the effect of the isolation kit were smaller than those of the sampling kit, indicating a more significant contribution of isolation kit to the overall model.

**Table 1:** Summary of taxa at all levels and detailed results for top 10 taxa significantly affected by sampling or DNA isolation kit.

Taxonomic level (# of all and significantly affected taxa)	Taxa (show ten most abundant)	q-value		Sign of the estimated effect size of the isolation or sampling kit				Relative abundance: total sum %	Gram stain
		isolation kit effect	sampling kit effect	PS to QS	SK2 to SK1	SK3 to SK1	SK3 to SK2		
**Phylum** All taxa: 14 Max >1% taxa: 6 Significantly affected taxa: 6 Isolation only: 1 Sampling only: 0 Both: 5	*Firmicutes*	**1.27E-15**	**4.43E-11**	+	−	−	+	68.3	G+
	*Bacteroidetes*	**3.42E-02**	**1.81E-02**	−	+	+	+	18.5	G−
	*Actinobacteria*	**3.67E-17**	**5.13E-04**	+	−	−	−	7.1	G+
	*Proteobacteria*	**5.05E-08**	**3.47E-04**	−	+	+	+	1.1	G−
	*Verrucomicrobia*	**1.69E-03**	**2.39E-03**	−	−	−	+	0.5	G−
	*Tenericutes*	**2.08E-03**	4.05E-01	−	−	−	−	0.1	G−
**Class** All taxa: 30 Max >1% taxa: 12 Significantly affected taxa: 12 Isolation only: 1 Sampling only: 0 Both: 11	*F; Clostridia*	**2.66E-15**	**9.45E-12**	+	−	−	+	61.2	G+
	*B; Bacteroidia*	**1.02E-02**	**2.15E-02**	−	+	+	+	18.5	G−
	*A; Actinobacteria*	**5.80E-14**	**3.04E-04**	+	−	−	−	4.6	G+
	*F; Negativicutes*	**2.58E-15**	**3.52E-04**	−	−	−	−	2.9	G−/var
	*A; Coriobacteria*	**5.80E-14**	**4.09E-03**	+	−	−	−	2	G+
	*F; Erysipelotrichia*	**2.66E-15**	**8.57E-05**	+	−	−	+	1.8	G+
	*F; Bacilli*	**2.58E-15**	**3.61E-05**	+	−	−	+	1	G+
	*V; Verrucomicrobiae*	**1.94E-03**	**1.33E-06**	−	−	−	+	0.5	G−
	*P; Betaproteobacteria*	**2.14E-05**	**1.49E-09**	−	+	+	+	0.4	G−
	*P; Gammaproteobacteria*	**3.67E-07**	**7.22E-03**	−	−	−	+	0.2	G−
**Order** All taxa: 49 Max >1% taxa: 13 Significantly affected taxa: 13 Isolation only: 1 Sampling only: 0 Both: 12	*F; Clostridiales*	**9.46E-13**	**1.37E-11**	+	−	−	+	61.2	G+
	*B; Bacteroidales*	**4.58E-03**	**1.69E-02**	−	+	+	+	18.5	G−
	*A; Bifidobacteriales*	**5.63E-12**	**2.30E-03**	+	−	−	−	4.5	G+
	*F; Selenomonadales*	**9.08E-17**	**2.42E-03**	−	−	−	+	2.9	G−/var
	*A; Coriobacteriales*	**5.73E-12**	**2.30E-02**	+	−	−	−	2	G+
	*F; Erysipelotrichales*	**3.05E-13**	**2.16E-04**	+	−	−	+	1.8	G+
	*F; Lactobacillales*	**3.05E-13**	**1.98E-04**	+	−	−	+	1	G+
	*V; Verrucomicrobiales*	**3.29E-04**	**1.22E-05**	−	−	−	+	0.5	G−
	*P; Burkholderiales*	**1.33E-05**	**1.70E-09**	−	+	+	+	0.4	G−
	*T; Mollicutes*	**1.04E-05**	**6.43E-05**	−	−	−	+	0.1	G−
**Family** All taxa: 85 Max >1% taxa: 23 Significantly affected taxa: 22 Isolation only: 5 Sampling only: 2 Both: 15	*F; Ruminococcaceae*	**9.20E-01**	**6.81E-13**	−	+	+	+	27.1	G+
	*F; Lachnospiraceae*	**5.68E-20**	**1.60E-03**	+	+	+	+	25.3	G+
	*B; Bacteroidaceae*	**7.55E-03**	**1.35E-02**	−	+	+	+	10.2	G−
	*A; Bifidobacteriaceae*	**5.90E-11**	**6.87E-03**	+	+	+	+	4.5	G+
	*F; Veillonellaceae*	**1.90E-12**	**1.55E-04**	−	+	+	+	2.4	G+
	*A; Coriobacteriaceae*	**7.68E-11**	**4.62E-02**	−	+	+	+	2	G+
	*F; Erysipelotrichaceae*	**1.74E-12**	**8.08E-04**	−	+	+	+	1.8	G+
	*F; Christensenellaceae*	**8.43E-01**	**1.24E-08**	−	+	+	+	1.4	G−
	*B; Rikenellaceae*	**5.90E-11**	7.24E-01	−	+	+	+	1.3	G−
	*B; Porphyromonadaceae*	**5.57E-04**	**4.03E-03**	−	+	+	+	1.1	G−
**Genus** All taxa: 277 Max >1% taxa: 82 Significantly affected taxa: 74 Isolation only: 27 Sampling only: 9 Both: 38	*B; Bacteroides*	**6.18E-03**	**1.54E-02**	−	+	+	+	10.2	G−
	*F; Faecalibacterium*	**1.37E-02**	**9.70E-05**	+	−	−	+	7.2	G+
	*F; Blautia*	**1.24E-24**	1.05E-01	+	−	−	+	5	G+
	*A; Bifidobacterium*	**1.48E-10**	**2.48E-02**	+	−	−	−	4.5	G+
	*F; Subdoligranulum*	**4.64E-03**	3.18E-01	−	−	−	+	3.7	G−
	*F; Pseudobutyrivibrio*	**9.63E-10**	6.32E-01	+	−	−	+	2.8	G−
	*F; Dialister*	**3.17E-09**	**5.86E-03**	−	−	−	+	2.2	G−
	*F; Roseburia*	**1.85E-02**	4.95E-01	+	−	−	+	1.5	G+
	*A; Collinsella*	**8.59E-05**	3.91E-01	+	+	−	−	1.4	G+
	*F,Christensenellaceae R-7 group*	6.06E-01	**1.50E-07**	−	−	−	−	1.4	G−

The significant q - values are shown in bold. SK1- stool container; SK2 – flocked swabs; SK3 - cotton swabs; PS – PowerLyzer PowerSoil DNA Isolation Kit; QS - QIAamp DNA Stool Mini Kit. All taxa – number of taxa found at the respective taxa level; Max >1% taxa – number of taxa that fulfilled the selection criteria for the analysis; Significantly affected taxa – the overall number of taxa at the respective taxa level affected by the isolation or sampling kit; Isolation only – number of taxa at the respective taxa level affected by the isolation kit only; Sampling only – number of taxa at the respective taxa level affected by the sampling kit only; Both – number of taxa at the respective taxa level affected by both sampling and isolation kit.

**Figure 4:**
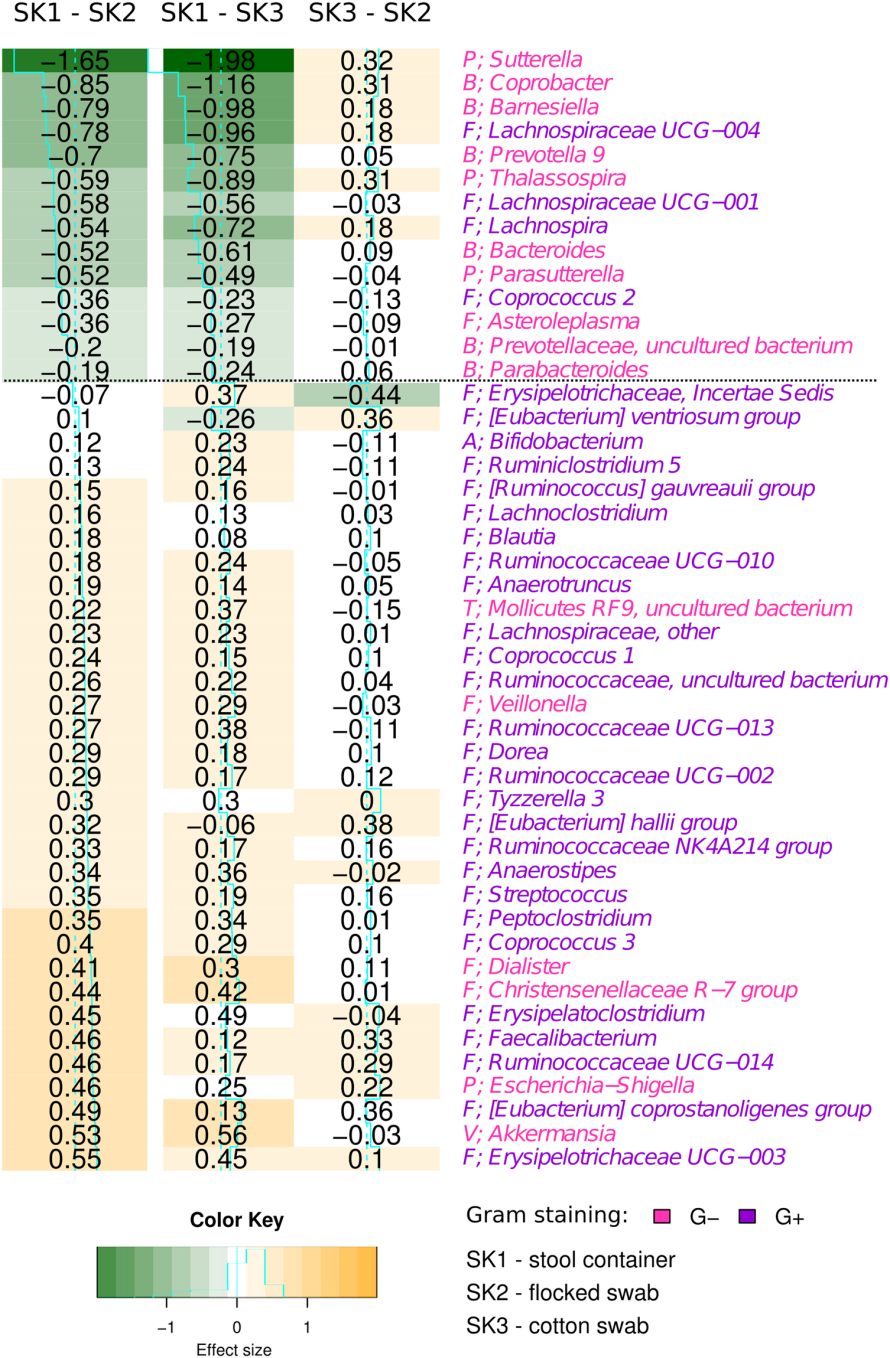
Comparison of sampling kits effects at genus level. Each column corresponds to a pair of sampling kits and each row corresponds to a specific bacteria genus. The values represent log fold changes of bacterial abundances (effect size) between the sampling kits, color coded from green (less abundant) to orange (more abundant). Only significantly affected taxa are shown.

**Figure 5:**
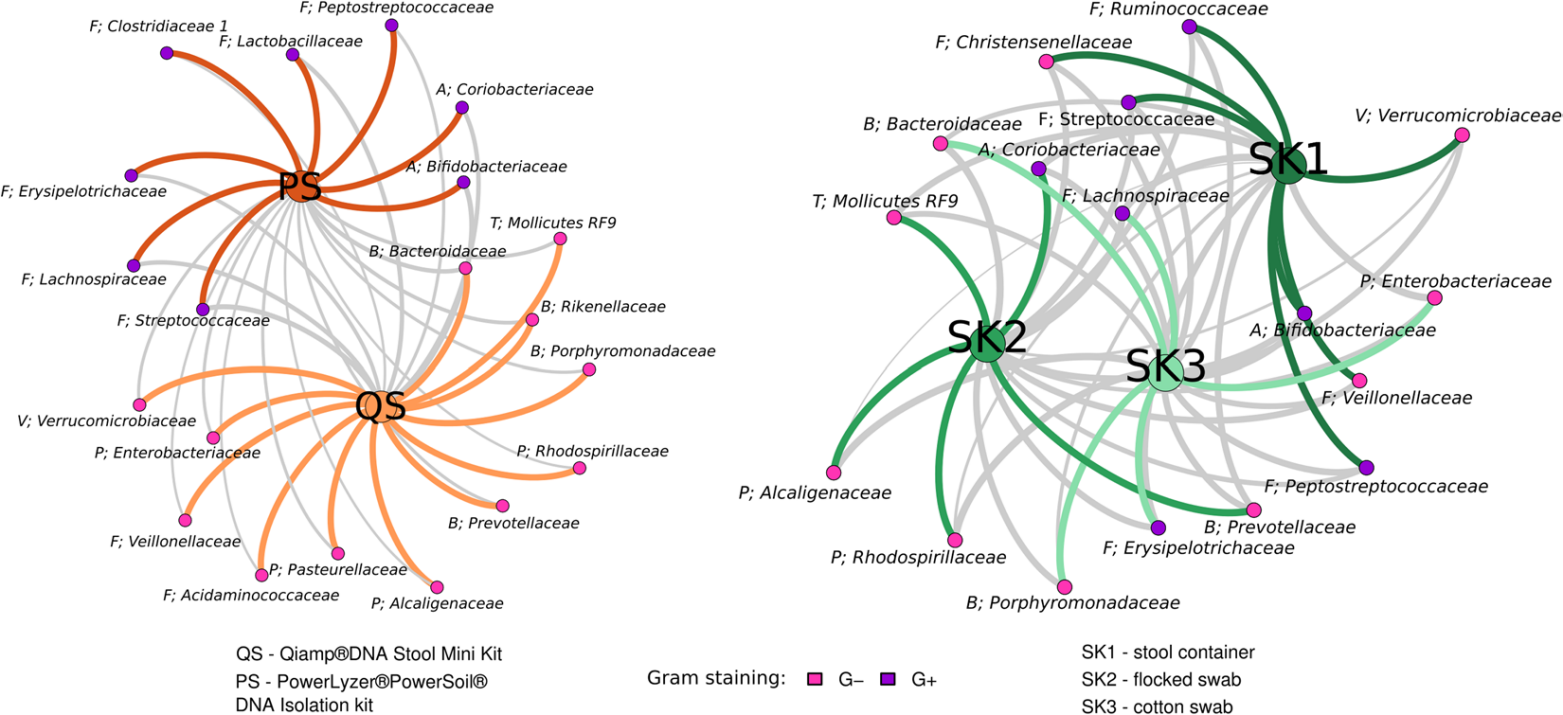
Association of bacterial families significantly differentially abundant between different sampling and isolation kits. The strength of the edges is weighted by relative abundance of taxa between the different kits (the stronger the edge, the larger the difference). Color-coding of the edges highlights taxa belonging to the same community, as detected by network modularity (see Methods for details). Grey edges represent connections between different communities.

We hypothesized that the observed effect of the isolation kit was a result of different efficiency of the kit-specific bacterial cell walls lysis procedure. In this case, one of the kits would be more successful in isolating Gram-positive (G+) bacterial species. The Table 2 shows the numbers of significantly affected G+ taxa in all taxonomic levels and statistical pairwise comparison of their proportion after both isolation methods and all sampling methods. We found significantly higher proportions of G+ bacteria after the isolation using the PS kit at all the taxon levels. (96.4% to 100%, Table 2), compared to the QS isolation kit (G+ proportion varying from 0 to 44%). Similar observations were made for the effect of the sampling kit (Table 2), but this trend was not significant on any of the taxa levels except for the comparison of cotton swab (SK2) and stool container (SK1) on the genus level. We hypothesize that these differences are attributed to the dilution of the samples during the preprocessing steps specific to the stool container (see Methods for more details), resulting in lower sample density thus increasing the efficiency of the bead beating procedure. No difference in proportion of Gram-positive bacteria was found between flocked and cotton swabs. Figure 4 shows estimated effect sizes pairwise between the sampling kits on the genus level. Figure 5 visualizes bacteria with significant changes in abundance between isolation or sampling kits, with nodes colored according to Gram-positivity, where we can observe association of Gram-positive bacteria with the PS isolation kit.

**Table 2:** Results of statistical testing of the proportion of G+ bacteria between significantly more abundant taxa within the selected isolation or sampling kit (pairwise).

		Phylum		Class		Order		Family		Genus	
Sample groups	Signif. more abundant	% of G+ phyla	q-val	% of G+ classes	q-val	% of G+ orders	q-val	% of G+ families	q-val	% of G+ geni	q-val
PS to QS	in PS	100% (2/2)	**6.67E-02**	100% (5/5)	**2.71E-03**	100% (5/5)	**2.10E-03**	100% (8/8)	**1.98E-05**	96.4% (27/28)	**1.98E-05**
	in QS	0% (0/4)		0% (0/6)		0% (0/7)		0% (0/12)		44.1% (15/34)	
SK2 to SK1	in S1	66.7% (2/3)	4.00E-01	71.4% (5/7)	2.27E-01	62.5% (5/8)	2.27E-01	58.3% (7/12)	1.10E-01	80.8% (21/26)	**4.29E-02**
	in S2	0% (0/2)		0% (0/3)		0% (0/3)		0% (0/5)		44.4% (4/12)	
SK3 to SK1	in S1	66.7% (2/3)	4.00E-01	71.4% (5/7)	2.27E-01	62.5% (5/8)	2.27E-01	58.3% (7/12)	1.10E-01	80.0% (20/25)	1.10E-01
	in S3	0% (0/2)		0% (0/3)		0% (0/3)		0% (0/5)		38.5% (5/13)	
SK3 to SK2	in S3	25% (1/4)	5.37E-01	37.5% (3/8)	5.37E-01	33.3% (3/9)	5.37E-01	35.7% (5/14)	5.37E-01	60.7% (17/28)	5.37E-01
	in S2	50% (1/1)		100% (2/2)		100% (2/2)		66.7% (2/3)		80.0% (8/10)	

The significant q – values are shown in bold. SK1- stool container; SK2 – flocked swabs; SK3 – cotton swabs; PS – PowerLyzer PowerSoil DNA Isolation Kit; QS – QIAamp DNA Stool Mini Kit. Sample groups – which pairwise comparison was performed; Signif. more abundant – in which group the taxa were significantly more abundant; % of G+ taxa – proportion of G+ in the significantly more abundant taxa within the respective group and level.

## Discussion

The gut microbiome seems to be crucial factor influencing human health and to date, a number of different diseases were correlated with microbiome dysbiosis. Understanding the true role of microbiome and fully comprehending its variability will require many cohort studies and, most probably, comparison of their results in large-scale meta-analyses. As with any other scientific domain, the incoherent methodological approaches constitute an important obstacle for such comparisons^44^. In an attempt to elucidate some of the factors determining the success of such studies, we focused on the effects of sampling and DNA extraction methods on a number of relevant variables from DNA integrity to final bacterial composition at different taxa levels. For this purpose, we selected sampling and DNA isolation kits that are the most common and accessible and hence are probably the most relevant for majority of cohort studies.

Our group of sixteen healthy volunteers used three different sampling kits – stool container, flocked swabs and cotton swabs. Without exception, the stool container was indicated as the most acceptable by the volunteers. Moreover, stool in the container can be easily diluted, homogenized and aliquoted for different analyses. Unfortunately, the stool container is inconvenient for sampling diarrhea or baby stool. Importantly, as we discuss below, the pre-processing specific to stool container samples influences both DNA quality and bacterial composition and these effects seem to interact with the DNA isolation kit.

For measuring the effect of different DNA extraction procedures, we used PowerLyzer PowerSoil DNA Isolation Kit (PS) and QIAamp DNA Stool Mini Kit (QS).

While the PS kit cell-wall lysis procedure is based on combination of bead-beating step and enzymatic lysis, the standard protocol of the QS kit comprises only enzymatic lysis. Considering the fact that the beat-beating step leads to higher DNA yield and higher number of observed OTUs from difficult-to-lyse bacteria, we added the bead-beating step also into the QS protocol, as commonly recommended^8,30,34,35,39^.

DNA isolation by the QS kit resulted in significantly higher DNA yields compared to the PS kit (regardless of the sampling kit). Similar results were observed in other studies^30,32^. In agreement with previous studies^30,35,37^, we found no significant correlation between DNA yield and alpha diversity.

On the other hand, the PS kit produced DNA of better integrity, even though in the PS protocol we applied more rigorous mechanical lysis (or higher speed of bead beating), which, according to the literature, should result in more degraded DNA^48^. We hypothesize that the observed differences might be caused by another factor, such as the type of the beads (0.1 mm glass in PS vs 0.1 mm zirconia in QS), the buffer composition, or the incubation temperature. Overall, for preparation of the shotgun libraries or sequencing using third generation of sequencers, we consider DNA integrity to be more important factor than the DNA yield, which favors PS kit over the QS kit.

To properly homogenize the samples from the stool container, we included a preprocessing procedure comprising five times dilution. This naturally resulted in lower yields of isolated DNA, but after adjustment for this dilution we obtained higher final DNA concentrations compared to undiluted stool samples from flocked and cotton swabs. It seems that the dilution step also affected the DNA integrity. Compared to the undiluted samples from flocked and cotton swabs, stool container samples resulted in less degraded DNA after isolation using the PS kit and, in contrast, in more degraded DNA after isolation using the QS kit. Interestingly, two other independent studies, where different isolation kits were used, showed either a negative^34^ or a positive^48^ effect of sample dilution on the DNA integrity. This, together with our results leads us to conclude, that the effect of dilution step on DNA integrity is dependent on the isolation kit.

PCR inhibitors persisted in the DNA of the samples after isolation with both kits. Presence of PCR inhibitors could complicate the use of conventional molecular methods for the detection of low abundance or rare pathogenic microorganisms^49,50^. The dilution of stool container samples prior to processing has led to significantly lower proportion of PCR inhibitors, hence for some applications, this approach might be preferred.

Both DNA extraction kits isolated preferentially bacterial DNA, independently on the sampling kit used and the amount of human DNA was negligible. From practical point of view, there is no superiority of any of the DNA isolation vs sampling kit combinations with respect to amount of residual human DNA. Some of the studies, however, use these kits to estimate the concentration of human DNA in stool samples as an indicator of inflammation that might predict onset of certain bowel diseases^51–55^. From this perspective, based on our results, we do not consider these kits eligible for human DNA quantification.

As for the alpha diversity, we observed increased number of OTUs after DNA isolation with the PS kit in all sampling kits, but the difference was significant only for cotton swab samples. We observed significant differences in number of OTUs between all sampling kits combinations, with the stool container resulting in the highest number of OTUs. We attribute the observed differences to higher effectivity of bead beating process in the less dense samples (the dilution preprocessing step used for the stool container). This is in contrast with the results of Santiago *et al*.^34^, who report no changes in alpha diversity after sample dilution. In that study, however, a different isolation kit was used, so the results are not directly comparable.

The final bacterial composition was more affected by the choice of the DNA isolation kit than by the choice of the sampling kit. The preference of the PS isolation kit for Gram-positive bacteria was confirmed by statistical testing on all taxa levels and we believe that it is a result of more effective lysis of the Gram-positive cell wall bacteria when using the PS kit, despite the additional bead-beating step we introduced into the QS protocol. This is in agreement with previously published results^8,26^. It has to be taken into account, that Gram staining not always corresponds with the cell wall structure (e.g. *Pseudobutyrivibrio*^56^ or *Deinococcus*^57^, which is for many bacteria unknown. The efficiency of the lysis procedure can be as well influenced by atypical composition of the cell wall, presence of S-layer or capsules. The bacterial cell wall type also plays a role in the sampling effect: in our study it was associated with the dilution preprocessing step of the stool container, although less significantly.

There is a common belief that the effect of the individual is the most influential on the final bacterial composition^8,32^. Indeed, many metagenomic studies are reporting differences between groups of interest at the OTU level, where the effect of isolation and sampling is less important, as we showed in this study. However, some hypotheses are connecting particular disease with higher or lower bacterial abundance at the phylum or family level. An example is the commonly used *Bacteroidetes*/*Firmicutes* ratio^58–64^. Our results show, that this ratio is very dependent on both the selected DNA isolation method and sampling kit (dilution step). In our study, the PS kit and the dilution step (stool container) led to significantly higher proportion of e.g. *Firmicutes* (G+) and *Actinobacteria* (G+) and significantly lower proportion of *Proteobacteria* (G−) and *Bacteroidetes* (G−).

Another example of the cell wall structure effect is the Gram-positive genus *Blautia*. *Blautia* is a common and highly prevalent bacteria in the gastrointestinal tract, which is connected with healthy gut, since it is an effective short-chain fatty acid producer^65,66^. Lower abundance of *Blautia* in the gut is associated with many diseases^66–73^. In our study, *Blautia* was bacteria the most significantly affected by DNA isolation (across all the taxonomic levels). Similar observations were also described as the effect of isolation in other studies^26,34^.

The sampling kit (dilution effect) influenced most significantly the abundance of genus *Sutterella*, bacteria correlated with many diseases such as celiac diseases^67^, Down syndrome^74^, autism^75^ or irritable bowel syndrome^76^. Clearly, the dilution step represents an important batch effect, which raises a question, whether it is related only to the artificial dilution, or this effect could also be observed in diarrheic samples. The effect of stool consistency was described previously as an important factor^12,77,78^ influencing the bacterial composition, but this effect was not connected with effect of higher water content (dilution), rather with the transit time. As previously recommended^77^, we also suggest to control for the stool consistency as a potential confounding factor to avoid the effect of sample water content in this kind of studies, especially if one of the illness symptoms is diarrhea.

Despite the fact that the significance of the sampling and isolation dependent batch effects is repeatedly reported, no systematic study of these effects was performed yet on samples from larger numbers of individuals. Efforts for standardization of laboratory practices in metagenomics have been made in large international projects such as Metagenomic Research Group (MGRG), Genomic Standard Consortium (GSC), The Microbiome Quality Control Project (MBQC) and International Human Microbiome Standards (IHMS). IHMS recommends a procedure for fecal sample DNA extraction, based on study of Costea *et al*., where 21 extraction protocols were compared, including protocols similar to ours – protocol 3 (with PowerLyzer PowerSoil DNA Isolation Kit) and 11 (with QIAamp DNA Stool Mini Kit and bead beating step)^39^. They selected the protocol with QIAamp DNA Stool Mini Kit as the best choice for its accuracy and reproducibility. In contrast to our results, both protocol 3 and 11, provide good lysis of Gram-positive bacteria, but protocol 3 was excluded for insufficient DNA quality. The main difference between the studies is that the Costea study was based on the results of whole metagenomics sequencing and only compared bacterial composition annotated at the species level.

All these above mentioned studies and our results confirm that meta-analytical studies are extremely challenging due to the many sources of batch effects that need to be accounted for. Incorporation of a standardized mock community to the sequencing workflow, followed by normalization of the results to these reference values could be solution in future. The increased cost per run and slightly more complex library preparation is a small price to pay for robustness, consistency and comparability of results.

## Conclusions

We performed systematic study of effects of DNA isolation and sampling kit on DNA quality and bacterial composition based on sequencing of gene for 16S rRNA on a the largest number of individuals to day (96 samples from 16 individuals).

We found significant effect of both DNA isolation and sampling kits on DNA purity, DNA integrity, alpha diversity and bacterial composition. Overall, the DNA isolation effect was stronger than that of the sampling kit. Interestingly the proportion of taxa affected by isolation or sampling was decreasing with decreasing taxonomical level.

We confirmed previously reported effect of DNA isolation kit on bacterial composition due to bacterial cell wall structure, namely the better efficacy of The PowerLyzer PowerSoil DNA Isolation Kit in lysis of Gram-positive bacteria. In addition, we report that the dilution pre-processing step of the stool container samples favored Gram-positive bacteria, although mostly at the genus level.

Both the choice of isolation and sampling kits significantly affected the *Firmicutes* to *Bacteroidetes* ratio. We conclude that the choice of DNA isolation and sampling kit (dilution step, and by extension the stool consistency) is an important batch effect that has to be taken into account mainly when comparing results between studies.

## Methods

### Sample collection

**Figure 6:**
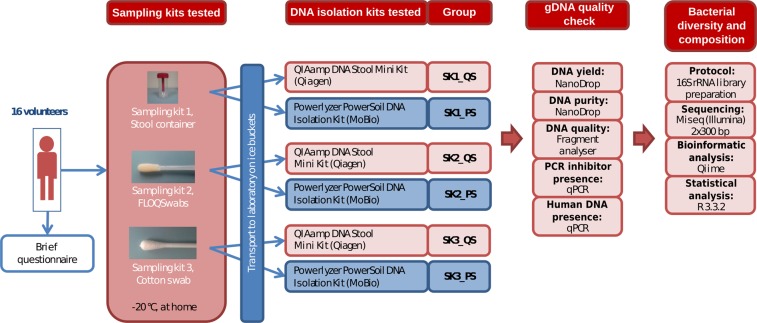
Study design. Flowchart summarizing the study design and methods used.

Stool samples were collected from a group of 16 volunteers. The subjects were 23–65 years old with an average age of 40.9 and none of them suffered from diarrhea during sample collection. Stool samples were collected at home. Volunteers received three stool sampling kits: sampling kit 1 (SK1) comprising 1x stool container (FL Medical, Italy); sampling kit 2 (SK2) comprising 2x flocked swabs (Copan, Italy) and sampling kit 3 (SK3) comprising 2x cotton swabs (SceneSafe, Great Britain). Sampling kits also contained disposable gloves and hand and surface disinfectant wipes for more convenient sampling. Each volunteer was instructed to collect all the samples from the same stool and from the same spot. Stool samples were then stored in a freezer at −20 °C overnight to freeze completely and the next day were transported on ice buckets to the laboratory, where they were stored at −20 °C prior to processing. Each group of samples was processed at the same time and by the same person. Participants filled out a brief questionnaire about satisfaction with individual sampling kits after stool sample collection. The study design is summarized in Fig. 6.

This study was carried out in accordance with the recommendations of the ELSPAC Steering Committee of Masaryk University with written informed consent from all subjects. All subjects gave written informed consent in accordance with the Declaration of Helsinki. The protocols were approved by the ELSPAC Steering Committee of Masaryk University.

### DNA extraction

Stool in the stool container (SK1) was diluted 5x with molecular grade water and homogenized by vortexing with Zirconia beads 2.3 mm (BioSpec, USA) to receive identical aliquots. This step is not necessary for the swabs, since each swab serves as an aliquot itself. Stool suspension (250 µl) was used for DNA extractions. Flocked swabs (SK2) and cotton swabs (SK3) were transferred into 2 ml tubes to be prepared for subsequent DNA extraction. DNA extractions were performed using a PowerLyzer PowerSoil DNA Isolation Kit (Mo Bio, USA) (PS) and QIAamp DNA Stool Mini Kit (Qiagen, USA) (QS) according to the manufacturer’s instructions.

Deviations from PS protocol:

- 750 µl of Bead Solution and 60 µl of C1 Solution were added to swab samples (SK2 and SK3) after defrosting. Samples were thoroughly vortexed and centrifuged for 4 min at 36,220 RCF. The swabs were then removed. Next, the samples were homogenized using the FastPrep-24 (MP Biomedicals, USA) 45 s 6.5 m/s.

Deviations from QS protocol:

- A homogenization step with 0.1 mm zirconia beads (BioSpec, USA) was added to the protocol after the third step (i.e. after the suspension was heated for 5 min at 95 °C).
- 1.4 µl Buffer ASL was added to swab samples (SK2 and SK3) after defrosting. Samples were vortexed continuously for 1 min and the suspension was heated for 5 min at 70 °C. Next, the samples were homogenized using the FastPrep-24 (MP Biomedicals, USA) 45 s 5.5 m/s.

### Evaluation of DNA yield, purity and quality

The final yield of extracted DNA was determined spectrophotometrically using theNanoDropND-1000 (Thermo Fisher SCIENTIFIC, USA). The purity of extracted DNA was indicated by an A260/A280 nm ratio. The quality of extracted DNA was assessed using the Fragment Analyzer (Advanced Analytical Technologies, USA) and High Sensitivity Genomic DNA Analysis Kit (Advanced Analytical Technologies, USA). The percentage of short fragments (≤1,500 bp) and Genomic Quality Number (GQN threshold of 10,000 bp) were calculated by PROSize 2.0 (Advanced Analytical Technologies, USA). Extracted DNA from each sample was diluted approximately to 5 ng/µl, aliquoted and stored at −20 °C. Aliquots were subsequently used in all further methods as starting material.

### Presence of PCR inhibitors after different DNA extractions

The presence of inhibitors was tested by qPCR. A primer pair specific for the conservative regions of 16S rRNA gene (Table 3) was used. qPCR was performed on the TOptical Thermocycler (Analytik Jena - Biometra, Ireland) using a KAPA SYBR FAST qPCR Kit (Kapa Biosystems, USA). Cycling conditions are described in Table 2. Melting temperature was determined after PCR to verify the correctness of each PCR product. Extracted DNA from four different isolates of *Escherichia coli* DH10B served as a positive control without PCR inhibitors. Each extracted DNA from sample and positive control (concentration approximately 5 ng/µl) was diluted three times (10x, 100x, 1,000x). The subsequent qPCR reactions were performed using both diluted and undiluted samples. Inhibition plots were created from Ct values and efficiency (=10^(−1/slope)^−1) was calculated for each sample and positive control.

**Table 3:** Primers and cycling conditions used in this study.

Target region/gene	Amplicon size	Primer name	Primer Sequences (5′ → 3′)	Cycling conditions						Reference
16S rRNA gene (bacterial DNA)	146 bp	q16S-univF	GTGSTGCAYGGYTGTCGTCA	95 °C	45x	95 °C	53 °C	72 °C		Maeda *et al*.^90^
		q16S-univR	ACGTCRTCCMCACCTTCCTC	10 min		20 s	30 s	20 s	—	
GAPDH (human DNA)	74 bp		TGCACCACCAACTGCTTAGC	95 °C	40x	95 °C	65 °C			Vandesompele *et al*.^91^
			GGCATGGACTGTGGTCATGAG	10 min		10 s	60 s	—	—	
V3/V4 16S rRNA gene (library preparation)	~460 bp	s16S_F	TCGTCGGCAGCGTCAGATGTGTATAAGAGACAG-InnerTag-CCTACGGGNGGCWGCAG	95 °C	25x	95 °C	55 °C	72 °C	72 °C	Klindworth *et al*.^79^
		s16S_R	GTCTCGTGGGCTCGGAGATGTGTATAAGAGACAG- InnerTag-GACTACHVGGGTATCTAATCC	3 min		30 s	30 s	30 s	5 min	

### Proportion of human DNA to bacterial DNA after different DNA extractions

The ratio of human and bacterial DNA in samples was tested by qPCR. Bacterial DNA was assessed using a primer pair specific for the conservative regions of 16S rRNA gene and human DNA using a primer pair specific for protein kinase (Table 3). qPCRwas performed on the TOptical Thermocycler (Analytik Jena - Biometra, Ireland) with KAPA SYBR FAST qPCR Kit (Kapa Biosystems, USA). Cycling conditions are described in Table 3. Melting temperature was determined after PCR to verify the correctness of each PCR product. The amount of human DNA to bacterial DNA was calculated as 2^ΔCt^. Ct value of 40 was used for all samples under the limit of detection.

### PCR amplification and Illumina library preparation

Extracted DNA was used as a template in amplicon PCR to target the hypervariable V3 and V4 regions of the bacterial 16S rRNA gene. The 16S metagenomics library was prepared according to the Illumina 16S Metagenomic sequencing Library Preparation protocol with some deviations described below (for workflow diagram see Supplementary Fig. S1). Each PCR was performed in triplicate, with the primer pair consisting of Illumina overhang nucleotide sequences, an inner tag and gene-specific sequences^79^. The Illumina overhang served to ligate the Illumina index and adapter. Each inner tag, i.e. a unique sequence of 7–9 bp, was designed to differentiate samples into groups. Primer sequences and PCR cycling conditions are summarized in Table 3. After PCR amplification, triplicates were pooled and the amplified PCR products were determined by gel electrophoresis. PCR clean-up was performed with Agencourt AMPure XP beads (Beckman Coulter Genomics, USA). Samples with different inner tags were equimolarly pooled based on fluorometrically measured concentration using Qubit dsDNA HS Assay Kit (Invitrogen, USA) and microplate reader Synergy Mx (BioTek, USA). Pools were used as a template for a second PCR with Nextera XT indexes (Illumina, USA). Differently indexed samples were quantified using the KAPA Library Quantification Complete Kit (Kapa Biosystems, USA) and equimolarly pooled according to the measured concentration. The prepared library was checked with a 2100 Bioanalyzer Instrument (Agilent Technologies, USA) and concentration was measured with qPCR shortly before sequencing. The library was diluted to a final concentration of 8 pM and 20% of PhiX DNA (Illumina, USA) was added. Sequencing was performed with the Miseq reagent kit V3 using a MiSeq. 2000 instrument according to the manufacturer’s instructions (Illumina, USA).

### Data analysis

Forward and reverse pair-end reads, that fulfilled the condition of both quality and length filtering, were merged using the fastq-join method within the join_pair_ends.py command in QIIME 1.9.1^80^. Data were demultiplexed and barcodes and primers were trimmed using package Biostrings^81^ in R 3.3.2^82^. Operational taxonomic units (OTUs) were constructed by binding sequences into clusters of greater than 97% sequence similarity using QIIME. In the next step, chimeras were detected on the set of representative sequences of each OTU with UCHIME in USEARCH v6.1.544^83^. These chimera OTUs were subsequently excluded from the analysis. Taxonomy was assigned to each OTU based on SILVA 123 reference database^84^. The observed species metric and the Chao1 index were used to estimate alpha diversity for each sample in QIIME. Beta diversity was computed in QIIME using both weighted and unweighted UniFrac metrics^85^. All statistical analysis was performed in R 3.3.2^82^.

The data were treated as compositional (proportions of total read count in each sample, non-rarefied) and prior to all statistical analyses were transformed using centered log-ratio transformation^86^. The analyses were performed on each of the seven taxonomy levels (Phylum, Class, Order, Family, Genus, Species and OTUs) separately and the resulting p-values were adjusted for multiple hypothesis testing using Benjamini-Hochberg procedure. Results were considered significant at FDR = 10%. The adjusted p-values are referred to as q-values.

To estimate the effects of isolation and sampling kits on bacterial composition while accounting for repeated measurements (effect of individual), we applied linear mixed model with sampling and izolation kits as fixed effects and individual as random effect (intercept). Log-likelihood test was performed to detect significance of each of the fixed effects – each time we compared the full model to the model without the fixed effect of interest.

A non-parametric Wilcoxon paired test, was used for comparison of effect of isolation kits on DNA quality. We used Spearman’s rank order correlation coefficient to discover the strength of the link between the number of observed species and DNA concentration.

Bipartite networks were used to visualize the influence of different kits on detection of Gram-positive and Gram-negative bacteria. These networks were reconstructed according to Sedlar *et al*.^87^ using R 3.3.2 and visualized in Gephi 0.9.2^88,89^. Communities within networks were extracted using modularity optimization criterion^88^.

## Supplementary information

Suppplementary Figure S1

Supplementary table S1

Supplementary table S2

Supplementary table S3

Supplementary table S4

Supplementary table S5

Supplementary table S6

Supplementary table S7

Supplementary table S8

Supplementary table S9

Supplementary table S10

Supplementary table S11

## Data Availability

Sequencing data were uploaded to the European Nucleotide Archive under accession number PRJEB24411. Read counts per sample at different taxa levels and sample information table are available in Supplementary Files S9–S11.
