# Supplementary material for: Stool sampling and DNA isolation kits affect DNA quality and bacterial composition following 16S rRNA gene sequencing using MiSeq Illumina platform
Source: Sci Rep. 2019 Sep 25;9:13837. doi: 10.1038/s41598-019-49520-3 (PMC6761292; doi:10.1038/s41598-019-49520-3)
Supplement: Supplementary file 1 — Suppplementary Figure S1 [file 41598_2019_49520_MOESM1_ESM.pdf]

Title page

Stool sampling and DNA isolation kits affect DNA quality and bacterial composition following 16S rRNA gene sequencing using MiSeq Illumina platform

Short title: Systematic assessment of stool sampling and DNA isolation kits.

Petra Videnska[1], Kristyna Smerkova[1], Barbora Zwinsova[1], Vlad Popovici[1], Lenka Micenkova[1], Karel Sedlar[2], Eva Budinska[1]*

[1]Research Centre for Toxic Compounds in the Environment, Masaryk University, Kamenice 5, 625 00 Brno, Czech Republic
[2]Department of Biomedical Engineering, Brno University of Technology, Technická 12, Brno, Czech Republic

videnska@recetox.muni.cz
k.smerkova@gmail.com
zwinsova@recetox.muni.cz
popovici@recetox.muni.cz
micenkova@recetox.muni.cz
sedlar@feec.vutbr.cz
budinska@recetox.muni.cz

*Corresponding author

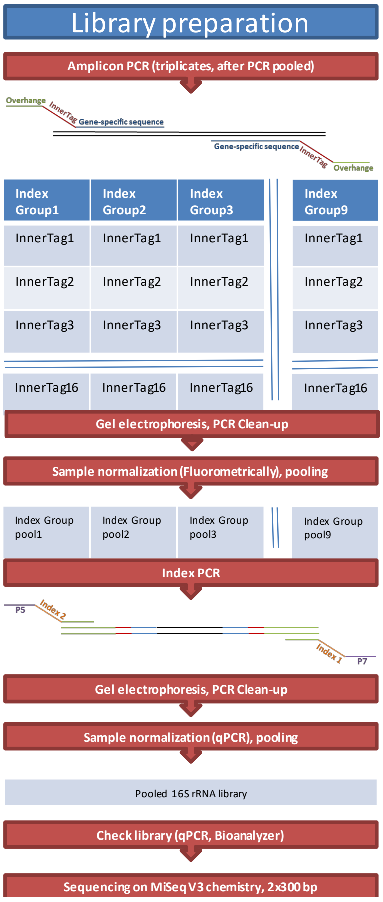

Supplementary figure 1: Workflow diagram of library preparation.
